# Therapeutic Potential of Polyphenols in the Management of Diabetic Neuropathy

**DOI:** 10.1155/2021/9940169

**Published:** 2021-05-13

**Authors:** Md. Tanvir Kabir, Nuzhat Tabassum, Md. Sahab Uddin, Faissal Aziz, Tapan Behl, Bijo Mathew, Md. Habibur Rahman, Raushanara Akter, Abdur Rauf, Lotfi Aleya

**Affiliations:** ^1^Department of Pharmacy, Brac University, Dhaka, Bangladesh; ^2^Department of Pharmacy, East West University, Dhaka, Bangladesh; ^3^Department of Pharmacy, Southeast University, Dhaka, Bangladesh; ^4^Pharmakon Neuroscience Research Network, Dhaka, Bangladesh; ^5^Laboratory of Water, Biodiversity & Climate Change, Cadi Ayyad University, Marrakech, Morocco; ^6^National Centre for Studies and Research on Water and Energy (CNEREE), Cadi Ayyad University, Marrakech, Morocco; ^7^Chitkara College of Pharmacy, Chitkara University, Rajpura, Punjab, India; ^8^Department of Pharmaceutical Chemistry, Amrita School of Pharmacy, Amrita Vishwa Vidyapeetham, AIMS Health Sciences Campus, Kochi 682041, India; ^9^Department of Chemistry, University of Swabi, Anbar, Swabi, Khyber Pakhtunkhwa, Pakistan; ^10^Chrono-Environnement Laboratory, UMR CNRS 6249, Bourgogne Franche-Comté University, Besançon, France

## Abstract

Diabetic neuropathy (DN) is a common and serious diabetes-associated complication that primarily takes place because of neuronal dysfunction in patients with diabetes. Use of current therapeutic agents in DN treatment is quite challenging because of their severe adverse effects. Therefore, there is an increased need of identifying new safe and effective therapeutic agents. DN complications are associated with poor glycemic control and metabolic imbalances, primarily oxidative stress (OS) and inflammation. Various mediators and signaling pathways such as glutamate pathway, activation of channels, trophic factors, inflammation, OS, advanced glycation end products, and polyol pathway have a significant contribution to the progression and pathogenesis of DN. It has been indicated that polyphenols have the potential to affect DN pathogenesis and could be used as potential alternative therapy. Several polyphenols including kolaviron, resveratrol, naringenin, quercetin, kaempferol, and curcumin have been administered in patients with DN. Furthermore, chlorogenic acid can provide protection against glutamate neurotoxicity via its hydrolysate, caffeoyl acid group, and caffeic acid through regulating the entry of calcium into neurons. Epigallocatechin-3-gallate treatment can protect motor neurons by regulating the glutamate level. It has been demonstrated that these polyphenols can be promising in combating DN-associated damaging pathways. In this article, we have summarized DN-associated metabolic pathways and clinical manifestations. Finally, we have also focused on the roles of polyphenols in the treatment of DN.

## 1. Introduction

Diabetic neuropathy (DN) is a common disorder and a microvascular complication of diabetes. Diabetic peripheral neuropathy (DPN) is linked with significant morbidity, mortality, and decreased quality of life [1]. The occurrence of neuropathy in diabetic individuals is around 30%, while up to 50% of diabetic individuals will develop neuropathy [2]. It has been estimated that around 472 million people will be affected by diabetes by 2030, while DPN will affect around 236 million individuals globally [3]. In general, DPN can be classified into focal/multifocal varieties and generalized polyneuropathies [4, 5]. Furthermore, this generalized form of DPN can also be divided into atypical and typical forms, depending on the alteration in onset, duration, pathophysiology, clinical manifestations, and associations. Indeed, typical DPN is a progressive and symmetrical length-dependent sensorimotor polyneuropathy.

In addition, typical DPN is the most common symptom of diabetes-related injury of the peripheral nervous system [6]. Since there is an increased rate of DN occurrence, it is important to study DN pathophysiology and therapeutic approaches in detail. DN can be developed on a hyperglycemia background and related metabolic imbalances, primarily oxidative stress (OS). Further complications can take place due to the hyperglycemia-mediated overgeneration of free radicals. Various studies have detected main pathways that are associated with DN, including the induced level of polyol, advanced generation of glycation end products, and other cascades of stress responses [7]. It has been identified that OS plays a crucial role in DN development.

Despite advances in the therapy of diabetes complications including DN, still, there is a deficiency of effective therapeutic agents. Furthermore, current drugs that are available to treat DN involve various common and serious adverse effects (Table 1). There is an increased need of developing novel multitarget therapeutic agents to control more destructive signaling mechanisms in patients with DN. It has been reported that polyphenols [8] are multitarget agents that exert effective antioxidant and anti-inflammatory properties. It has been confirmed by various *in vitro* and *in vivo* studies that natural phenolic compounds play important roles in the management of type 2 diabetes via insulin-dependent mechanisms (Table 2). Besides, polyphenols have the efficacy to fight against various diseases including diabetes and diabetes-associated complications [8–10]. Polyphenols have been reported to exert potent neuroprotective activities in case of diabetes [11].

In order to develop polyphenols as therapeutic agents to treat DN, it is essential to understand the signaling mechanisms that are associated with the advancement of DN and the mechanisms by which polyphenols avert the advancement of these destructive mechanisms. In this article, we have summarized metabolic pathways and clinical manifestations that are associated with DN. Moreover, we have also focused on the roles of polyphenols in the treatment of DN.

## 2. Diabetic Neuropathy Pathogenesis-Associated Metabolic Pathways

It has been revealed that various mechanisms are associated with the development of DN pathogenesis including imbalances in the blood supply to peripheral nerves, gene expression of calcium and sodium channels, vascular system of the thalamic gland, and autoimmune disorders characterized via glial cell activation [71]. The main mediators and signaling mechanisms that are linked with DN include glutamate pathway, activation of channels, trophic factors, inflammation, OS, advanced glycation end products (AGEs), and polyol pathway [72–76].

### 2.1. Glutamate Pathway

Glutamate is important for various processes including cell migration, cell death, cell differentiation, and synapse plasticity [77]. In the central nervous system (CNS), glutamate also has a significant contribution to the peripheral transduction of sensory inputs [78]. Multiple studies have revealed that glutamate-induced toxicity is present in case of both chronic and acute neurodegenerative disorders of the CNS and peripheral nervous system (PNS) [79]. It has been reported that glutamatergic ligands can induce nociceptive behaviors, which indicates that glutamate is associated with peripheral sensory transduction and nociceptive pathways. In a mouse model of type I diabetes, hyperglycemia markedly elevated the expression of N-methyl-D-aspartate (NMDA) receptors [80]. In addition, activities of spinal NMDARs have been confirmed in nerve injury-mediated pain [81]. Interestingly, spinal NMDAR subunit 2B (NR2B) level was increased in both protein and mRNA levels of STZ-mediated DN, which further resulted in hyperactivity of spinal cord dorsal horn neurons [82]. It has been observed that glutamate (particularly NR2B) induces various DN-associated pathways including apoptosis, inflammation, and OS [83]. In order to combat DN, targeting the glutamate pathway and NR2B as upstream factors of apoptotic, inflammatory, and oxidative mechanisms via phytochemicals is highly promising.

### 2.2. Activation of Channels

**Figure 1:**
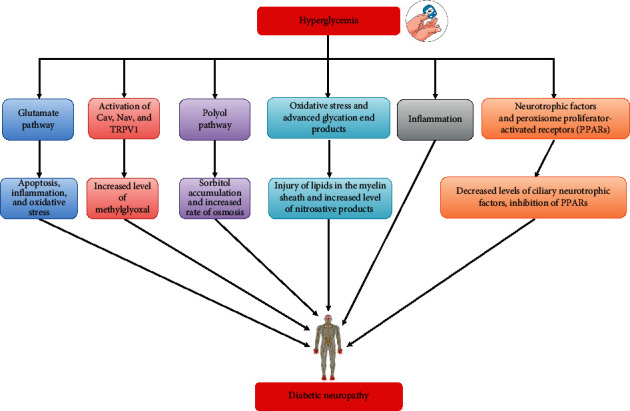
Diabetic neuropathy pathogenesis-associated metabolic pathways.

Transient receptor potential vanilloid 1 (TRPV1) channel is associated with multiple modalities of nociceptive stimuli. In a streptozotocin- (STZ-) mediated DN model, the expression of TRPV1 was markedly elevated in individuals with hyperalgesic skin in comparison with the individuals with hypoalgesic and normoalgesic skin [84, 85]. Various studies have revealed that early stages of DN take place because of the TRPV1 upregulation via protein kinase C (PKC) and protein kinase A (PKA) [86], which further indicates the contribution of TRPV1 channels in hyperalgesia expression [86]. In case of DN, other TRPV receptors are yet to be properly investigated [87]. TRPV may be generally considered as an auspicious therapeutic target to develop new therapeutic agents for DN. TRPV1 activation-induced [Ca^2+^] transients and found to be commonly altered in hyperalgesia [86]. Therefore, voltage-gated calcium channels (CaVs) are supposed to be associated with painful DN [88]. It has been reported that the *α*2*δ* subunits elevated the trafficking and expression of these channels, however might have a contribution to synaptogenesis within the CNS and PNS [89]. Along with CaVs, an increased level of voltage-gated sodium channels (Nav) was detected at the site of neuronal injury in DN [90]. Furthermore, an increased level of methylglyoxal has been identified in the serum of patients with painful DN. Methylglyoxal resulted in mechanical and thermal hyperalgesia when injected into the diabetic mouse models (but not in Nav1.8 knockout mouse models) [91]. Collectively, all these findings suggest the significance of CaVs, Nav, and TRPV1 via multitarget phytochemicals in the DN development (Figure 1). Besides, TRPV1 was found to be coexpressed with glutamate receptors [92].

### 2.3. Polyol Pathway

Polyol pathway exists in various tissues including blood vessels and peripheral nerve and plays significant roles in DN development [93]. There are two major enzymes that are associated with the polyol pathway including sorbitol dehydrogenase and aldose reductase (AR). These enzymes are found in several tissues including vascular cells, glomerulus, retina, lens, and nerve [94]. Increased levels of blood glucose can result in AR activation that generates sorbitol from glucose. Indeed, this reaction utilizes nicotinic acid adenine dinucleotide phosphate (NADPH) and generates NADP^+^. Increased NADPH utilization can decrease the concentration of a decreased level of glutathione (GSH) and elevates its oxidized form GSH disulfide. Since sorbitol is unable to cross the cell membrane, sorbitol accumulation increases blood osmolality which further results in the loss of electrolytes [74, 95]. Increased level of osmosis can lead to injury of cells that are located adjacent to peripheral neurons (Schwann cells) and results in a schwannopathy-associated phenotype of DN [95, 96]. It has been reported that sorbitol dehydrogenase can trigger the conversion of accrued sorbitol into fructose through oxidation and generation of nicotinic acid adenine dinucleotide. Nonetheless, increased levels of fructose and sorbitol exert harmful actions in nerve cells because of various causes including the reduced level of the osmolality regulator (taurine), regulator of insulin sensitivity (myoinositol), suppression of the Na^+^/K^+^ ATPase pump, intracellular Na^+^ accumulation, ionic homoeostasis through reducing the PKC effect which results in swelling of the axon and axon-glia dysfunction, and decreased level of nerve conduction velocity (NCV) [72].

It has been revealed that accumulated glucose can enter into the hexosamine biosynthesis pathway and generates fructose-6-phosphate, which gets eventually converted into uridine diphosphate-N-acetylglucosamine (GlcNAc). In addition, GlcNAc is a sugar moiety that is utilized in O- or N-glycosylation of such translated proteins (posttranslational modification) since SP-1 transcription factor results in plasminogen activator inhibitor-1 overexpression and growth factor-*β*1 transformation. Therefore, these factors result in nerve injury by generating mitochondrial superoxides [97]. In animal models, inhibitors of AR were found to be very effective in reducing DN [98]. However, these inhibitors were not that much effective in clinical studies [99], which was partial because of the introduction of lower doses as compared to *in vivo* studies. Thus, inadequate levels were available to avert the flux through the polyol pathway [98].

### 2.4. Oxidative Stress and Advanced Glycation End Products

In case of OS, oxidation surpasses antioxidant ability in cells because of the imbalance in the level of enzymatic antioxidant catalase (CAT) and superoxide dismutase (SOD) or nonenzymatic factor GSH [100]. In the polyol pathway, NADPH consumption results in negative action in a decreased level of GSH. Increased level of reactive oxygen species (ROS) including hydrogen peroxide (H_2_O_2_), hydroxyl radical (^•^OH), and superoxide (O_2_^•–^) was found to damage the proteins and lipid of cells. Furthermore, ROS can result in injury of lipids in the myelin sheath [101]. In a study, Edwards et al. [102] revealed that increased concentration of nitrosative products including nitrotyrosine (NT) and peroxynitrite (ONOO^−^) in diabetic individuals is positively associated with DPN. Therefore, the increased lipid peroxidation, and damage rate of DNA and protein. It has been reported that nonenzymatic reactions between the damaged lipids, DNA, or proteins and aldehyde groups of reducing sugars lead to AGEs, which further stimulate ROS generation both during their formation and interaction with the AGE receptor (RAGE) [103, 104]. In addition, advanced lipoxidation end products are generated via an elevated level of OS-stimulated lipid peroxidation along with altered lipid metabolism [105].

Other enzymes including PKC-*β*, 12/15-lipoxygenase, Na^+^/H^+^ exchanger, and NADPH oxidase are also associated with ROS generation in DN individuals [106, 107]. Indeed, PKC-*β* has a contribution to nerve activity and DN pathogenesis [108]. Interestingly, streptozotocin- (STZ-) induced diabetic rat models revealed the positive outcomes of the PKC-*β* inhibitor on DN in decreasing free radicals [109]. In case of hyperglycemia, the mitochondrial membrane's potential is disturbed, and it secretes cytochrome c which then causes activation of procaspase-9 along with apoptotic protease activating factor-1 (Apaf-1) resulting in the caspase-3 activation in neurons [110, 111]. In a STZ-induced rat model of diabetes, Zherebitskaya et al. [112] revealed that an increased level of glucose reduced manganese-containing superoxide dismutase (MnSOD) and elevated level of ROS in axons which predominantly resulted in the injury of dystrophic structures and axon outgrowth. Collectively, these findings regarding OS indicate that regulating the level of ROS in patients with diabetes might be a possible way of preventing DN.

### 2.5. Inflammation

Inflammation is the response which is activated via damage in the dorsal root ganglion (DRG), spinal cord, skin, or nerve, which eventually leads to painful sensation. Furthermore, it is linked with diabetes and increased concentrations of inflammatory cytokines including tumor necrosis factor-*α* (TNF-*α*) and C-reactive protein in individuals with DN [113]. In a study, Conti et al. [114] observed that STZ-induced diabetes resulted in the infiltration of immune cells including monocytes and macrophages, the neuronal overexpression of interleukin-1 beta (IL-1*β*), and the expression of neurotrophin receptor p75 [114]. Moreover, the association of inflammation in DN was demonstrated in a STZ-induced diabetic animal model. It was observed that pioglitazone reduced the level of phosphorylated extracellular signal-regulated kinases (ERKs), changed the protein kinase C-alpha expression level, and reduced the number of accumulated macrophages in Schwann cells [115]. Since a transcriptional factor comprises 2 subunits including p50 and p65, nuclear factor kappa B (NF-*κ*B) is located in the cytoplasm in an inhibitory state bound to the inhibitor of nuclear factor-*κ*B (I*κ*B).

After the simulation, I*κ*B is tagged through ubiquitin for proteasomal degradation leaving active NF-*κ*B. In the active state, NF-*κ*B is translocated to the nucleus, where it induces the expression of various survival and inflammatory genes. It has been revealed that the level of NF-*κ*B's p65 subunit is increased in the myelin sheath of neurons in case of demyelinating polyneuropathies [116]. In a different study, Ha et al. [117] revealed that hyperglycemia in glial cells induced NF-*κ*B activation, which further resulted in increased concentration of various cell adhesion genes and inflammatory genes (TNF-*α*, cyclooxygenase-2 (COX-2), inducible nitric oxide synthase, IL-1*β*, and interleukin 6 (IL-6)). In another study, Bierhaus et al. [118] detected IL-6, receptor for AGEs, and p65 subunit of NF-*κ*B in sural nerve biopsies obtained from people with diabetes. Collectively, these results confirmed that inflammatory signaling pathways have a significant contribution in DN pathogenesis which makes them important pharmacological targets via phytochemicals (Figure 1).

### 2.6. Neurotrophic Factors and Peroxisome Proliferator-Activated Receptors

Neurotrophic factors induce nerve regeneration, mediate normal physiological activities of surviving neurons, and also elevate their resistance to damage. These activities ameliorate the clinical conditions of patients with DN [119]. It has been observed that deficiency of neurotrophin plays a role in DN pathogenesis. Interestingly, the levels of ciliary neurotrophic factor, insulin-like growth factors, neurotrophin-3/4/5, and brain-derived neurotrophic factor (BDNF) were decreased in the muscles of patients with DN [120]. Indeed, nerve growth factor (NGF) can decrease these neurotrophin imbalances [119]. Preclinical experiments supported the idea that influencing neurotrophic factors via phytochemicals may be a potent therapeutic strategy for various types of peripheral nerve disease.

It is known that peroxisome proliferator-activated receptors (PPARs) are a group of nuclear receptor proteins. By binding with the lipophilic stimulant, PPAR mediates the expression of proximal genes that are associated with hepatocarcinogenesis, lipid hemostasis, proximal proliferation, and beta-oxidation of fatty acids [121–123]. There are 3 major subtypes of PPARs including *α*, *β*/*δ*, and *γ* that have a significant contribution in regulating inflammatory processes, morphogenesis, glucose, mobilization of lipids, storage, and metabolism [122, 124, 125]. PPARs work together with various cellular transcription factors including activated protein-1 (AP-1), signal transducer/activator of transcription-1, and NF-*κ*B [125]. Furthermore, PPARs suppress the expressions of chemokines and proinflammatory genes (interleukin 1 beta (IL-1*β*) and TNF-*α*) and decrease the sensation of pain [126]. Agonists of PPAR gamma (PPAR-*γ*) including rosiglitazone and pioglitazone are commonly suggested to treat insulin resistance and hyperglycemia [127] and to reduce the activation of spinal nociceptive neurons in type II diabetic rat models [128]. Furthermore, PPAR agonists have the future potential to be used as novel analgesics in treating various chronic pain conditions including DN. Nevertheless, their possible adverse effects need to be carefully considered during targeting PPAR signaling pathway as analgesics [129]. Thus, there is a growing research interest regarding the use of PPAR agonists to decrease DN.

## 3. Clinical Manifestations of Diabetic Neuropathy

DPN might present with various clinical signs and symptoms. In some cases, patients might be completely asymptomatic; however, foot ulcer might be the first presentation. Nevertheless, other individuals might exhibit one or multiple different symptoms including numbness, paresthesia, and neuropathic pain (frequently stated as aching, shooting, lancinating, or burning) which may range from mild to severe, which can lead to severe suffering [130]. In addition, these symptoms might be constant or sporadic. Interestingly, sensory symptoms might persist for a short duration before they vanish completely, or they might become chronic.

Sensory signs and symptoms first appear in the distal foot/toes. Pinprick and light touch of the distal foot is normally impaired on first physical examination and then more advanced motor (particularly loss of muscle bulk, loss of ankle reflex, clawing of the toes, and weakness) and sensory (specifically proprioception loss and vibration) abnormalities. It spreads proximally up the leg before affecting the upper limbs and fingertips as the disease advances. Physical examination for individuals with painful DPN is typically unclear as compared to those without neuropathic pain. Nonetheless, certain people might contain pure small fiber neuropathy, which can lead to a loss of small fiber modalities (particularly pinprick and loss of temperature sensation) with normal large fiber activity [6]. It was observed that a small number of individuals possess the so-called “irritable nociceptor” phenotype with “positive” sensory signs including hyperalgesia and allodynia [131, 132].

## 4. Polyphenols in the Treatment of Diabetic Neuropathy

**Figure 2:**
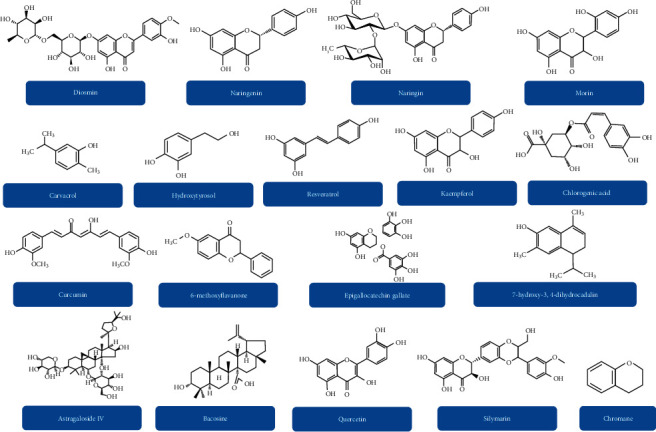
Chemical structures of various polyphenolic compounds that can be effective in the treatment of diabetic neuropathy.

It has been reported that around 800 plants might contain antidiabetic properties. So far, various phytochemicals including kolaviron, resveratrol, naringenin, quercetin, kaempferol, and curcumin (Figure 2) have been administered in patients with DN. However, it is essential to detect the phytochemicals that can be used in the treatment of DN. In the following sections, we have summarized the cellular signaling pathways and pharmacological targets that are associated with the therapeutic effect of polyphenols in DN.

### 4.1. Nonenzymatic and Enzymatic Antioxidant Performance

In diabetic animals, hyperglycemia decreases the effect of antioxidant enzymes with nonenzymatic glycosylation and results in OS [133]. In DN development, stimulation of some negative effects including generation of free radicals by OS, lower GSH levels, Cu/Zn SOD, glutathione S-transferase, decreased glutathione peroxidase (GPx), oxidations of leukocytes and catecholamines, elevated mitochondrial leak, perglycemia, and ischemia play a major destructive role [133–136]. Various antioxidants, particularly polyphenols, have exerted some promising activities in the experimental DN treatment. In experimental DN, *α*-lipoic acid treatment averted neurovascular irregularities. In diabetic rat models, this treatment also attenuated GSH levels, digital nerve conduction velocity, and nerve blood flow via increasing free radical scavenging activity [137, 138]. Probucol is a strong free radical scavenger and an inhibitor of low-density lipoprotein oxidation that normalizes both electrophysiology and nerve blood flow [139]. In a study, Al-Rejaie et al. [140] revealed that naringenin contains antioxidant properties. Moreover, it inhibited the levels of nitric oxide (NO) and thiobarbituric acid reactive substances (TBARS) and attenuated the decreased concentrations of GPx and CAT in STZ-induced diabetic rat models [140, 141].

Resveratrol (a polyphenolic compound) (Figure 2) protected neural tissues from diabetes-mediated OS via decreasing the levels of malondialdehyde (MDA), xanthine oxidase (XO), and NO in the brain stem, spinal cord, cortex, hippocampus, and cerebellum via increasing the level of GSH in diabetic rat models [142]. In addition, apocynin and curcumin attenuated the elevated spinal H_2_O_2_ level and level of MDA and increased the level of SOD in STZ-induced diabetic rat models. It has been confirmed that curcumin suppressed the activation of spinal NADPH oxidases, the major enzymes that generate ROS via reversing the upregulation of phagocyte NADPH oxidase subunits (gp91^phox^ and p47^phox^) [143]. More related cellular signaling pathways and pharmacological targets that are associated with the antioxidant property of polyphenols have been summarized in Table 3.

### 4.2. Prevention of the Inflammatory Response and Proinflammatory Cytokines

Proinflammatory alterations that are seen in diabetes have a significant contribution to the pathogenesis of retinopathy, nephropathy, neuropathy, and atherosclerosis [175]. Production of hyperglycemia-mediated ROS is directly associated with the DN pathogenesis. Indeed, these ROS might trigger the generation of IL-1*β* and TNF-*α*. In the CNS, insulin resistance and hyperglycemia are linked with the TNF-*α* signaling pathway, which might trigger pain and hyperalgesia in DN [176–178]. Various studies have revealed that suppression of TNF-*α* decreased hyperalgesia in models of painful DN [179]. It has been confirmed that TNF-*α* intraplantar injection is linked with thermal hyperalgesia and mechanical allodynia in rat models [74, 176, 180]. IL-1*β* can be obtained from various cell types including Schwann cells, endothelial cells, mononuclear cells, synoviocytes, and fibroblasts and has a significant contribution in triggering mechanical hyperalgesia. In the mouse model of experimental neuropathy, it neutralized the IL-1 receptors that further resulted in the reduction of pain-related behavior [181, 182].

**Figure 3:**
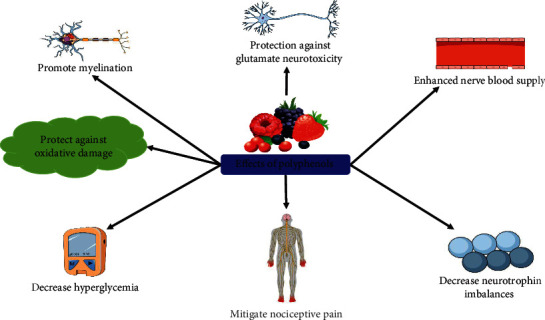
Possible effects of polyphenols in the management of diabetic neuropathy.

Increased lipid level and hyperglycemia resulted in activation of NF-*κ*B that has a significant contribution to the generation of ROS and TNF-*α*, which further induces inflammatory demyelination. NF-*κ*B might increase metabolic disorders including diabetes and trigger inflammation [183]. Inhibitor of NF-*κ*B (I*κ*B-*α*) and p65 are the subunits of NF-*κ*B that are overexpressed in sural nerve macrophages in chronic and acute inflammatory demyelinating polyneuropathies [116, 184]. In STZ-induced DN rat models, it has been confirmed that resveratrol exerts anti-inflammatory property via reducing the expression of I*κ*B-*α* and p65 and ameliorating the increased concentrations of NF-*κ*B, IL-6, COX-2, and TNF-*α* [169]. Furthermore, resveratrol markedly reduced the atherogenic index, serum glucose level, and expression of cerebral COX-2 and MDA [171]. In a study, Deng et al. [147] revealed that carvacrol reduced STZ-mediated DN by reducing the level of NF-*κ*B p65 subunit, TNF-*α*, caspase-3, and IL-1*β* [147]. Kaempferia (a polyphenol) decreased STZ-induced DN via reducing the levels of TNF-*α* and IL-1*β* and inhibiting the formalin-triggered nociceptive behavior (Figure 3). Moreover, it improved lipopolysaccharide-mediated inflammatory mediators (such as ROS, IL-1*β*, TNF-*α*, phagocytosis, prostaglandins, and NO) in microglial cells [162, 185].

### 4.3. Antinociceptive Activities

Hyperglycemia-induced ROS generation and lipid peroxidation in sciatic nerves decreased endoneurial blood flow and induced sciatic nerve dysfunctions in case of DN. Indeed, neuropathic pain is a common diabetes-associated complication that takes place due to the induction of the abnormal activity of the CNS or PNS, which further leads to central sensitization, alterations of primary afferent nerves, and sensory abnormalities. Various studies have already confirmed the efficacy of tramadol, dextromethorphan, lamotrigine, phenytoin, pregabalin, gabapentin, tricyclic antidepressants (TCAs), gamma-aminobutyric acid (GABA), and opioids in the treatment of painful sensory neuropathy. Even though these therapeutic agents may relieve the pain by 30 to 50%, their uses are often limited because of marked side effects [175]. Thus, there is an increased need of using polyphenols as alternative therapies. In a plantar heat hyperalgesia test, quercetin (Figure 2) significantly suppressed the increase of paw withdrawal threshold (PWT) in STZ-induced diabetic rat models which was assessed through Hargreaves' test. On a Randall–Selitto paw pressure device, quercetin also markedly elevated mechanical PWT as compared to STZ-induced diabetic control rats [141]. In addition to this, quercetin elevated the tail withdrawal latency in both nondiabetic and diabetic mouse models [167]. In a dose-dependent manner, it also has markedly elevated the paw and tail withdrawal latency and reduced the number of foot slips of STZ-induced diabetic rat models in comparison with the normal control [154, 165, 186].

As compared to the control group, Kaur et al. [161] revealed that chromane markedly corrected the reduced PWT of STZ-induced diabetic rat models in hot-plate and tail immersion tests [161]. In diabetic rat models, 6-methoxyflavanones and chlorogenic acid elevated mechanical and thermal PWT, respectively [157, 158]. In a different study, in STZ-induced diabetic rat models, Attia et al. [150] showed that combined administration of gabapentin and curcumin resulted in a marked rise in mechanical PWT along with tail-flick and hot-plate latencies. Interestingly, curcumin significantly elevated the pain threshold, reaction times, and tail-flick latencies [148]. As compared to untreated diabetic rat models, curcumin treatment increased the antinociceptive effect in hot-plate and allodynia tests in STZ-induced DN by elevating the pain threshold [149]. It has been reported that diosmin and oryzanol markedly elevated the tail-flick latency in the tail immersion test and decreased thermal hyperalgesia in STZ-induced diabetes [163]. In diabetic rat models, treatment with diosmin also markedly ameliorated the shortening of time on walking function tests [144]. In a study, Kumar et al. [169] confirmed that resveratrol treatment significantly corrected the reduction of PWT and tail-flick latency in hot and cold immersion performance [169]. In addition, two polyphenols including 7-hydroxy-3,4-dihydrocadalin and silymarin resulted in a marked reduction of pain scores of the formalin test [159, 173].

### 4.4. Enhancement of Nerve Growth Factors

Multiple complex processes are associated with DN including various molecular changes and sensory modalities. In the nervous system, various neurotrophic factors (especially NGF) affect the population of certain neurons [118]. DN might be regulated via neurotrophins including transient receptor potential ion channels, such as vanilloid receptor 1 and NGF, including its receptors p75 and tyrosine kinase A (TrkA) and their downstream signaling pathways. NGF exerts significant neuroprotective activity, and it causes axonal growth. Indeed, pathological conditions that change NGF levels can induce neurons to lose their activity and die. Following nerve injury and inflammation, NGF level elevates in the nervous system and mediates pain and hyperalgesia that can be decreased via anti-NGF therapy. Interestingly, the complex of TrkA and NGF sensitizes VR1 thereby elevating pain. After the binding of NGF with TrkA, several processes including cell survival, nerve regeneration, and neurite growth pathways will start [187]. IGF-1 is structurally similar to insulin, and it has a significant contribution to cellular growth and proliferation. IGF-1 is also a potent apoptosis inhibitor. In addition, it regulates the development and growth of DNA synthesis and nerve cells [174]. In morin-treated diabetic animals, levels of IGF-1 and NGF in sciatic nerves significantly increased as compared to the negative control group [174].

In diabetic rat models, Methycobal and mulberry flavonoids lessened the inhibition of the average optical density of the myelin sheath and myelinated extramedullary fiber cross-sectional area. Interestingly, animals pretreated with 0.3 g/kg mulberry flavonoids exhibited ultrastructural properties of myelin, significantly decreased the level of myelin breakdown, and also caused significant axonal improvement [160]. In diabetic rat models, astragaloside IV (a polyphenol) inhibited a reduction in the myelinated fiber area and density and segmental demyelination via reducing the levels of AR and hemoglobin A1c (HbA1C) in erythrocytes, which further increased the plasma insulin concentrations and GPx function in nerves. In STZ-induced diabetic rat models, astragaloside IV increased Na^+^/K^+^-ATPase activity in both erythrocytes and nerves [152]. Treatment with grape seed proanthocyanidins ameliorated the abnormal activity of the peripheral nerve and impaired nerve tissues. It also decreased the level of nerve conduction velocity (NCV) and the level of free Ca^2+^, which further increased the activity of Ca^2+^-ATPase in sciatic nerves [155]. In diabetic rat models, curcumin treatment gradually recovered cyclooxygenase function in the sciatic nerve [149].

### 4.5. Glutamate Pathway and NMDA Receptors

In experimental DN models, glutamate receptors and ligands are supposed to be associated with nociceptive behaviors. Besides, it is regarded that glutamate and N-methyl-D-aspartate (NMDA) receptors are associated with peripheral sensory transduction and nociceptive pathways [188]. NMDA receptors also couple with mitogen-activated protein kinase (MAPK) and extracellular signal-regulated kinase (ERK) phosphorylation and activation in the superficial laminae of the spinal cord that could be inhibited via treatment with NMDA receptor antagonists [83, 189, 190]. NMDA receptor ion channel-induced entry of calcium has a significant contribution to the activation of extracellular signal-regulated kinase (ERK) and MAPK pathways in painful DN [188]. It has been reported that resveratrol prevented glutamate injuries via blocking the NMDA receptor and inhibited glutamatergic neurotransmission [191, 192]. It also markedly reduced glutamine expression, transportation, synthetase to avert diabetic retinopathy [193]. Resveratrol also suppressed impairments in Na^+^/K^+^-ATPase, intracellular ROS generation, mitochondrial dysfunction, and activation of microglia [194, 195]. Furthermore, it decreased the level of glutamate-induced tissue plasminogen activator through ERK and AMPK/mammalian target of rapamycin signaling pathways and reduced the activation of MAPK, which eventually inhibited the activity of the voltage-dependent Ca^2+^ channel and suppressed induced release of glutamate [196, 197]. Like resveratrol, piceatannol stimulated the expression of nuclear factor erythroid 2-related factor 2-dependent and heme oxygenase-1 and thus protected HT22 neuronal cells from glutamate-mediated cell death [198].

Chlorogenic acid (a polyphenol) protected against glutamate neurotoxicity via its hydrolysate, caffeoyl acid group, and caffeic acid via controlling the entry of calcium into neurons [199, 200]. Epigallocatechin-3-gallate mediated protection of motor neurons which was found to be linked with the regulation of glutamate concentration [201]. Furthermore, it suppressed glutamate dehydrogenase in pancreatic *β*-cells and activated adenosine monophosphate-activated protein kinase to positively influence diabetes [202]. Like quercetin, epigallocatechin-3-gallate also decreased glutamate-mediated raised level of calcium via attenuating PKC and influx of ionotropic Ca^2+^ [203–205]. In diabetic rat models, curcumin prevented intracellular elevation of calcium [206], improved both NR2B gene expression and glutamate level [207, 208], and attenuated excitotoxicity mediated by the NMDA receptor [209]. It also influenced the PI3K/AKT signaling pathway and downstream signaling pathways via BDNF and TrK*β*, perhaps via reducing the activation of MAPK/ERK [210, 211]. It was revealed that naringin, chlorogenic acid, and apigenin 8-C-glucoside control glutamate pathways [212, 213]. Kaempferol and astragaloside IV attenuated OS and glutamate-induced toxicity [214, 215]. Therefore, these polyphenols might be good options in preventing complications related to DN (Figure 2).

## 5. Future Research Directions

DN is one of the most distressing diabetes-associated complications that affects over 30% of diabetic people worldwide. In addition, there are increasing types of diabetes-mediated peripheral nerve damages including mononeuritis multiplex, mononeuropathy, radiculopathy, diabetic amyotrophy, and autonomic and small fiber neuropathy. DN pathogenesis is multifactorial, and its main categories include ischemic and metabolic. In the treatment of neuropathic pain, even though opioid therapy and neuromodulating drugs, including anticonvulsants and TCAs, are effective treatments, these treatments are expensive. Because of the lack of safe and consistently effective therapies for DN, there is an increased need to develop novel herbal therapies to ameliorate the quality of life of DN individuals [216]. Various findings suggested that polyphenols exert protective activities by anti-inflammatory and antioxidant pathways. Indeed, polyphenols have the potential to fight against various chronic diseases including diabetes and diabetes-associated complications with less toxic effects in *in vitro* and animal models [9, 10]. In nutrition, these aforementioned properties have made polyphenols a promising area of research interest [217, 218].

## 6. Conclusion

Polyphenols are potent natural compounds that can be useful to combat DN via influencing various signaling mechanisms with fewer side effects. It has been confirmed that herbal therapies with various polyphenols can exert a positive effect on DN management. Further research regarding novel pathogenicity signaling mechanisms of DN, safety, and efficacy of polyphenols in humans may reveal more effective applications of polyphenols in the management, prevention, and treatment of DN. Nonetheless, more studies are required to develop more effective therapeutic agents for DN.

## Figures and Tables

**Table 1:** Common and serious adverse effects of currently available drugs that are used in the treatment of diabetic neuropathy.

Drug	Common adverse effects	Serious adverse effects	References
Amitriptyline	Nausea, insomnia, headache, blurred vision, dizziness, sedation, dry mouth, orthostatic hypotension, urinary retention	Hyponatraemia, serotonin syndrome, suicidal thoughts, hepatotoxicity, seizures, cardiac arrhythmias, interstitial lung disease	[12–14]
Gabapentin	Dry mouth, peripheral oedema, somnolence, gait disturbance, weight gain, headache, dizziness	Suicidal thoughts and behavior, Stevens–Johnson syndrome, seizures, hepatitis, withdrawal reactions, confusion	[15]
Tramadol	Nausea, headache, dizziness, sweating, constipation, somnolence	Hallucinations, seizures, opioid abuse/misuse, serotonin syndrome	[16]
Duloxetine	Dizziness, nausea, headache, dry mouth, diarrhoea, somnolence, sweating, insomnia, constipation, tremor	Hepatic failure, serotonin syndrome, hypertensive crisis, urinary retention, interstitial lung disease, hyponatraemia, Stevens–Johnson syndrome, seizures	[17–19]
Venlafaxine	Nausea, headache, insomnia, vomiting, diarrhoea, sweating, dry mouth, anorexia, somnolence		[20]
Pregabalin	Dry mouth, dizziness, somnolence, weight gain, weakness, headache, peripheral oedema	Seizures, angioedema, hepatotoxicity, rhabdomyolysis, Stevens–Johnson syndrome, suicidal thoughts, cardiac arrhythmia, pulmonary oedema, thrombocytopenia	[21, 22]
Tapentadol extended release (ER)	Nausea, headache, somnolence, dizziness, sweating, constipation	Same as tramadol and angioedema	[23–26]

**Table 2:** Antidiabetic properties of naturally occurring phenolic compounds.

Naturally occurring phenolic compounds	Effects	References
Flavonoids	(i) Intestinal microbiota ↑ (ii) Digestive enzymes ↓ (iii) Glucose absorption ↓ (iv) Formation of advanced glycation end products (AGEs) ↓	[27–31]
Catechins	(i) Insulin sensitivity ↑ (ii) Fecal excretion of bile acids and cholesterol ↑ (iii) Activation of AMPK ↑ (iv) White fat depots ↓ (v) Blood lipid ↓ (vi) Glycaemia ↓ (vii) Pancreatic *α*-glucosidase and also *α*-amylase and maltase ↓ (viii) Generation of reactive oxygen species ↓ (ix) Na^+^-dependent glucose transporter ↓	[32–38]
Caffeoylquinic acids	(i) Insulin response ↑ (ii) Hepatic glucose-6-phosphatase ↓ (iii) Human pancreatic and salivary *α*-amylase ↓	[39–43]
Isoflavones	(i) Hypoglycemic effects through ameliorating insulin resistance and sensitivity ↑ (ii) Exerting anti-inflammation property ↑ (iii) Digestion of carbohydrate and uptake of glucose in the small intestine ↓ (iv) Protecting pancreatic *β*-cells ↑ (v) The mechanism of renal interstitial fibrosis in diabetic nephropathic rat models ↓ (vi) Oxidative damage ↓ (vii) Maillard reaction and formation of AGEs ↓	[44–46]
Hydroxycinnamic acids	(i) Insulin resistance and glucose intolerance ↑ (ii) Glucokinase activity ↑ (iii) *β*-Cell activity ↑ (iv) Antioxidant properties and anti-inflammatory activities ↑ (v) Activation of AMP-activated protein kinase ↑ (vi) Phosphoenolpyruvate carboxykinase and glucose-6-phosphatase effects in the liver ↓ (vii) Gluconeogenesis and adipogenesis ↓	[47–50]
Stilbenoids	(i) Metabolic control ↑ (ii) Pancreatic *β*-cell and hepatoprotective activity ↑ (iii) Insulin sensitivity ↑ (iv) DNA integrity ↑ (v) Level of digestive enzymes ↓ (vi) Oxidative stress and inflammation ↓	[51–57]
Tannins	(i) Uptake of glucose in adipose tissue through phosphorylation of IRS-1 ↑ (ii) Phosphorylation of AMPK ↑ (iii) Formation of AGEs and enzymatic action of sucrose, lactase, and maltase ↓ (iv) Activities of *α*-amylase and *α*-glucosidase ↓	[35, 58, 59]
Procyanidins	(i) Target digestive enzymes ↓ (ii) AMPK and insulin signaling pathways ↑ (iii) Cellular expression of NAD^+^ and SIRT1 levels ↑ (iv) Proinflammatory cytokine expression ↓	[60–63]
Anthocyanins and anthocyanidins	(i) Antioxidant ↑ (ii) Blood glucose regulation ↑ (iii) Anti-inflammatory activity ↑ (iv) Oxidative damage ↓ (v) Concentrations of cholesterol, low-density cholesterol, and triglycerides ↓	[64–67]
Curcumin	(i) Protecting pancreatic *β*-cells ↑ (ii) Diabetic cardiomyopathy ↓ (iii) Insulin resistance ↓ (iv) Oxidative damage ↓	[44, 68–70]

Note: ↑ = induction; ↓ = inhibition.

**Table 3:** Polyphenols in the treatment of diabetic neuropathy.

Polyphenols	Animal models	Duration	Dosage	Effects	References
Diosmin	STZ-induced diabetic rats	4 weeks	50 and 100 mg/kg/day	Increased tail-flick latency; decreased traveling duration; increased concentration of SOD and GSH; decreased levels of MDA and NO	[144]
Hydroxytyrosol	STZ-induced diabetic rats	6 weeks	10 and 100 mg/kg/day	Reduced thermal nociception; elevated paw withdrawal threshold and Na^+^/K^+^ ATPase activity; increased MNCV level	[145]
Kolaviron	STZ-induced diabetic rats	6 weeks	100 and 200 mg/kg/day	Reduced level of OS, IL-1*β*, TNF-*α*, MDA, and TBARS; elevated concentrations of GSH, CAT, and GPx	[146]
Carvacrol	STZ-induced diabetic rats	7 weeks	25, 50, and 100 mg/kg/day	Increased SOD level; decreased concentrations of IL-1*β*, MDA, and TNF-*α*	[147]
Naringenin	STZ-induced diabetic rats	5 weeks	25 and 50 mg/kg/day	Increased tail-flick latency and paw withdrawal; elevated NGF and IGF-1 in sciatic nerves; decreased IL-1*β* and TNF-*α* levels; increased concentrations of CAT, GSH, and GPx	[140]
Naringin	STZ-induced diabetic rats	4 weeks	40 and 80 mg/kg/day	Reduced mechanotactile allodynia, oxidative-nitrosative stress, and TNF-*α* level; increased tail-flick latency and nociceptive threshold; increased concentrations of MNCV and SOD	[141]
Curcumin	STZ-induced diabetic rats	6 weeks	100 mg/kg/day	Decreased thermal nociception, levels of TNF-*α* and IL-10; increased tail-flick latency and paw withdrawal threshold	[148]
	STZ-induced diabetic rats	3 weeks	200 mg/kg/day	Decreased mechanical allodynia and thermal hyperalgesia; increased paw withdrawal threshold; decreased AR, prostaglandin peroxidase, and COX levels	[149]
	STZ-induced diabetic rats	14 days	200 mg/kg/day	Increased paw withdrawal threshold and SOD level; decreased levels of MDA and H_2_O_2_ in the spinal cord	[143]
Curcumin and gliclazide	STZ-induced diabetic rats	5 weeks	100 mg/kg/day	Increased mechanical hyperalgesia threshold, hot-plate, and tail-flick latencies; decreased levels of peroxynitrite, LPO, and TNF-*α*	[150]
Curcumin and resveratrol	STZ-induced diabetic rats	4 weeks	Curcumin = 60 mg/kg/day; resveratrol = 20 mg/kg/day	Increased nociceptive threshold; decreased levels of brain nitrite and TNF-*α*	[151]
Astragaloside IV	STZ-induced diabetic rats	12 weeks	3, 6, and 12 mg/kg/day	Increased myelinated fiber density, myelinated fiber area, and segmental demyelination; decreased levels of HbA1C; increased levels of MNCV and GPx; decreased AR level in erythrocytes; increased activity of Na^+^/K^+^ ATPase in nerves and erythrocytes	[152]
Epigallocatechin gallate	STZ-induced diabetic rats	10 weeks	2 g/L/day	Decreased mechanical allodynia and thermal hyperalgesia; elevated paw withdrawal pressure; decreased 8-OHdG immunoreaction, numbers of Fos-immunoreacted neurons, and colocalization of 8-OHdG and Fos in laminae I–III	[153]
	STZ-induced diabetic rats	7 weeks	20 and 40 mg/kg/day	Increased nociceptive threshold and tail-flick latency; reduced formalin-mediated nociceptive behavior; decreased concentrations of nitrite, TBARS, and MDA; elevated SOD level	[154]
Grape seed proanthocyanidins	STZ-induced diabetic rats	16 weeks	125, 250, and 500 mg/kg/day	Increased hot-plate latency and nerve conduction velocity; decreased level of free Ca^2+^; elevated activities of ATPase in sciatic nerves	[155]
Bacosine	STZ-induced diabetic rats	30 days	5 and 10 mg/kg/day	Diabetes-linked cognitive impairment; decreased hyperalgesia; increased levels of MNCV and SOD; decreased levels of AGEs, ROS, MDA, TNF-*α*, and IL-1*β*	[156]
6-Methoxyflavanone	STZ-induced diabetic rats	—	10 and 30 mg/kg/day	Elevated paw withdrawal threshold and latency; reduced thermal nociception; involvement of GABA receptors; increased flinching response threshold and latency by a preference for the *δ*- and *ĸ*-opioid receptors	[157]
Chlorogenic acid	STZ-induced diabetic rats	14 days	100 mg/kg/day	Increased threshold of mechanical hyperalgesia; decreased formalin-mediated nociceptive behavior	[158]
7-Hydroxy-3,4-dihydrocadalin	STZ-induced diabetic rats and mice	—	0.3–30 and 30–300 mg/kg/day	Decreased mechanical hyperalgesia and allodynia and formalin-evoked hyperalgesia; increased withdrawal threshold; reduced level of MDA	[159]
Mulberry flavonoids	ALX-induced diabetic rats	8 weeks	0.3 and 0.1 g/kg/day	Reduced myelin breakdown and myelinated fiber cross-sectional area; decreased peripheral nerve injury and numbers of extramedullary fiber of sciatic nerves	[160]
Chromane	STZ-induced diabetic rats	30 days	5 and 10 mg/kg/day	Decreased mechanical allodynia and thermal hyperalgesia; increased paw withdrawal threshold and MNCV level; decreased levels of AGEs and ROS	[161]
Kaempferol	STZ-induced diabetic mice	3 weeks	25, 50, and 100 mg/kg/day	Decreased formalin-mediated nociceptive behavior in phases 1 and 2 and oedema size; reduced hyperalgesia; elevated thermal pain threshold; decreased levels of IL-1*β*, TNF-*α*, LPO, and nitrite	[162]
Oryzanol	STZ-induced diabetic rats	—	50 and 100 mg/kg/day	Increased pain threshold, hot-plate latency, and GSH; decreased flinching in diabetic rats during both quiescent phase and phase 2 but not in phase 1; reduced nitrite and MDA levels; attenuated activity of Na^+^-K^+^ ATPase	[163]
Pepino polyphenolic extract	STZ-induced diabetic mice	12 weeks	—	Decreased concentrations of IL-6, TNF-*α*, AGEs, and ROS; increased GSH and GPx levels; elevated fascicle with numerous small myelinated fibers	[164]
Quercetin	STZ-induced diabetic rats	2 weeks	40 mg/kg/day	Increased hot-plate, tail-withdrawal latency, and cold allodynia latency; reduced number of foot slips	[165]
	STZ-induced diabetic rats	8 weeks	10, 20, and 40 mg/kg/day	Reduced thermal hyperalgesia and mechanical allodynia; increased concentrations of MNCV, SOD, and GPx; decreased levels of TNF-*α* and IL-1*β*	[166]
	STZ-induced diabetic rats	4 weeks	10 mg/kg/day	Increased tail-flick latencies and nociceptive threshold in both diabetic and nondiabetic mice	[167]
	STZ-induced diabetic rats	4 weeks	10 mg/kg/day	Decreased thermal nociception; elevated tail withdrawal latencies and nociceptive threshold	[168]
Resveratrol	STZ-induced diabetic rats	2 weeks	10 and 20 mg/kg/day	Increased tail-flick latency and paw withdrawal pressure; elevated concentrations of MNCV and CAT; reduced MDA level	[169]
	STZ-induced diabetic rats	2 weeks	10 and 20 mg/kg/day	Increased MNCV level; decreased concentrations of p65, MDA, NF-*κ*B, I*κ*B-*α*, TNF-*α*, IL-6, and COX-2	[170]
	STZ-induced diabetic rats	6 weeks	20 mg/kg/day	Decreased cerebral MDA and COX-2; increased cerebral level of IL-4 and GSH	[171]
	STZ-induced diabetic rats	>6 weeks	10 mg/kg/day	Decreased concentrations of MDA, XO, and NO; increased level of GSH in the cortex, hippocampus, brain stem, cerebellum, and spinal cord	[142]
	STZ-induced diabetic rats	2 weeks	20 mg/kg/day	Elevated tail withdrawal threshold and latencies	[172]
Silymarin	STZ-induced diabetic rats	8 weeks	100 and 200 mg/kg/day	Elevated tail-flick latency; decreased nociceptive scores in both phases of the formalin test	[173]
Morin	STZ-induced diabetic rats	3 weeks	15 and 30 mg/kg/day	Increased paw withdrawal and tail-flick latency; elevated NGF and IGF-1 in sciatic nerves; decreased levels of IL-1*β*, TNF-*α*, and LPO	[174]

8-OHdG: 8-hydroxy-2′-deoxyguanosine; AGEs: advanced glycation end products; ALX: alloxan; AR: aldose reductase; CAT: catalase; COX: cyclooxygenase; DNP: diabetic neuropathy; GABA: gamma-aminobutyric acid; GPx: glutathione peroxidase; GSH: glutathione; HbA1C: hemoglobin A1c; IGF-1: insulin-like growth factor; IL-10: interleukin-10; IL-1*β*: interleukin 1 beta; LPO: lipid peroxidation; MDA: malondialdehyde; MNCV: motor nerve conduction velocity; NGF: nerve growth factor; NO: nitric oxide; NOS: nitric oxide synthase; OS: oxidative stress; ROS: reactive oxygen species; SOD: superoxide dismutase; STZ: streptozotocin; TBARS: thiobarbituric acid reactive substances; TNF-*α*: tumor necrosis factor-*α*; XO: xanthine oxidase.
